# Selective Inhibition of Histone Deacetylation in Melanoma Increases Targeted Gene Delivery by a Bacteriophage Viral Vector

**DOI:** 10.3390/cancers10040125

**Published:** 2018-04-21

**Authors:** Samuel Campbell, Keittisak Suwan, Sajee Waramit, Eric Ofori Aboagye, Amin Hajitou

**Affiliations:** 1 Cancer Phage Therapy Laboratory, Division of Brain Sciences, Burlington Danes Building, Hammersmith Hospital Campus, Imperial College London, Du Cane Road, London W12 0NN, UK; samuel.campbell@zoo.ox.ac.uk (S.C.); Keittisak.suwan@imperial.ac.uk (K.S.); s.waramit15@imperial.ac.uk (S.W.); 2 Comprehensive Cancer Imaging Centre, Faculty of Medicine, Hammersmith Hospital Campus, Imperial College London, Du Cane Road, London W12 0NN, UK; eric.aboagye@imperial.ac.uk

**Keywords:** bacteriophage, C1A, targeted cancer gene therapy, HDAC inhibitors, HDAC6

## Abstract

The previously developed adeno-associated virus/phage (AAVP) vector, a hybrid between M13 bacteriophage (phage) viruses that infect bacteria only and human Adeno-Associated Virus (AAV), is a promising tool in targeted gene therapy against cancer. AAVP can be administered systemically and made tissue specific through the use of ligand-directed targeting. Cancer cells and tumor-associated blood vessels overexpress the α_ν_ integrin receptors, which are involved in tumor angiogenesis and tumor invasion. AAVP is targeted to these integrins via a double cyclic RGD4C ligand displayed on the phage capsid. Nevertheless, there remain significant host-defense hurdles to the use of AAVP in targeted gene delivery and subsequently in gene therapy. We previously reported that histone deacetylation in cancer constitutes a barrier to AAVP. Herein, to improve AAVP-mediated gene delivery to cancer cells, we combined the vector with selective adjuvant chemicals that inhibit specific histone deacetylases (HDAC). We examined the effects of the HDAC inhibitor C1A that mainly targets HDAC6 and compared this to sodium butyrate, a pan-HDAC inhibitor with broad spectrum HDAC inhibition. We tested the effects on melanoma, known for HDAC6 up-regulation, and compared this side by side with a normal human kidney HEK293 cell line. Varying concentrations were tested to determine cytotoxic levels as well as effects on AAVP gene delivery. We report that the HDAC inhibitor C1A increased AAVP-mediated transgene expression by up to ~9-fold. These findings indicate that selective HDAC inhibition is a promising adjuvant treatment for increasing the therapeutic value of AAVP.

## 1. Introduction

The risk of cancer appears to be an almost inevitable hazard associated with multicellularity and the need to replace cells over time, while also maintaining the ability to evolve through the accumulation of mutations. The latest World Health Organization (WHO) World Cancer Report (2014) reported that in 2012 there were 14.1 million new cancer cases, 8.2 million cancer deaths, and 32.6 million people living with cancer within five years of their diagnosis [1]. The WHO further predicts a 70% increase in cancer incidence over the next two decades [1].

When cancer is confined to a single location, the tumor can be removed surgically. However, if the cancer does not form a solid tumor (e.g., leukemia), or if it is located in a relatively inaccessible or critical region like the brain, where surgical removal of tissue can harm the patient independently of the cancer, then mechanical excision is often not an option. Even in cases where a solid tumor has been removed before clinically evident metastasis, it is impossible to confirm that every cancer cell has been mechanically excised, which often necessitates the use of systemic treatments, such as chemotherapy or radiotherapy. Both therapies broadly target rapidly dividing cells, which includes the bulk of cancer cells, but also impact numerous healthy tissues. The side effects caused by the destruction of healthy cells, particularly those of the gastrointestinal tract, are a commonly cited reason for non-compliance with self-administered chemotherapy regimens [2].

Even when treatment regimens are followed, heterogeneity in cancer subpopulations means that some cancer cells may have escape mutations, which can lead to the recurrence of a new generation of cancer cells resistant to the previously administered treatment [3]. Tragically, both chemotherapies and radiotherapies can be double edged swords, particularly in children, infrequently causing secondary malignant neoplasms [4,5]. For large swathes of the global population, these concerns are rendered moot, as systemic treatment is largely inaccessible or entirely unavailable in many countries. In these places, the incidence and mortality of cancer are nearly the same [1], emphasizing the importance of systemic treatments as well as the demand for treatments that can be manufactured at a lower cost.

The difficulties of fighting an evolutionary arms race against cancers, and the negative side effects of current systemic treatment methods, intensely underscore the desperate need for targeted systemic treatments, with less collateral damage to healthy tissues. Gene therapy, the delivery of functional nucleic acids to a cell, offers the possibility of an alternative form of systemic treatment. The ideas behind gene therapy in general are not new, proposed in the 1960s after the development of a gene transfer system in bacteria [6]; it was already specifically suggested in 1966 that viruses could be used to deliver genes as a treatment for cancer [7].

However, like any emergent field, gene therapy has suffered setbacks, such as the death of treatment subjects in 1999 and 2000, due to complications with adenoviral vectors and murine leukemia virus (MuLV)-derived vectors respectively [8]. Both trials used mammalian viruses with pathogenic wild type strains. There has been a shift away from vectors based on adenovirus and MuLV towards those based on lentiviruses and adeno-associated virus (AAV). Although lentiviruses are also retroviruses, proponents argue that their integration profiles are sufficiently different so as to allay the concerns surrounding MuLV [8]. Wild type AAV is not known to be associated with any human pathologies, and recombinant AAV is often entirely devoid of the viral genome other than sequences from the inverted terminal repeats (ITRs). One of the primary impediments to the use of AAV is economic, as it remains relatively expensive to produce.

A previously developed hybrid between AAV and an M13-derived filamentous bacteriophage, dubbed AAV-phage or AAVP, neatly tackles both the concerns of cross reactivity of mammalian viruses as well as the economic impediments for mass production of AAV [9]. AAVP is a bacteriophage containing an AAV transgene cassette, inserted within the phage genome and packaged by the phage capsid, and in which the native *rep* and *cap* genes have been replaced by a cytomegalovirus promoter, a transgene of interest, and a poly-adenylated tail. Using the well-established practice of phage display, a double cyclic arginyl glycyl aspartic acid peptide (CDCRGDCFC), dubbed RGD4C, was attached to the phage pIII minor coat protein. The RGD4C has been shown to target α_v_ integrins (principally α_v_β3 and α_v_β5 heterodimers) that are overexpressed in cancer cells and the irregular vasculature formed by cancer induced angiogenesis [10,11]. Because AAVP is manufactured in bacteria, as opposed to human cells like standard AAV, its production can be scaled up to large bioreactors with comparatively cheap inputs. The transgene cassette forms episomes in the host nucleus, rather than integrating into the host chromosome, avoiding concerns around proto-oncogene activation. The episomes are not replicated with cell division, so accidental uptake by non-targeted cells is eliminated over time.

Since bacteria are separated from humans by at least 3 billion years of evolution, phage have no natural tropism for mammalian cells. This allows targeting to be tightly controlled by humans via the attachment of ligands to phage capsid to bind mammalian receptors. AAVP with RGD4C targets cancer cells and endothelial cells supplying solid tumors, but other ligands can be used to target different cell types [12]. Ideally, this precision targeting makes AAVP a safe therapeutic approach against cancer compared to traditional treatments. This lack of tropism also means that the phage particles lack many of the pathogen-associated molecular patterns that are easily recognized by the mammalian immune system, and phage does not incite as robust an immune response upon first contact [13]. Phage viruses are not completely ignored by the mammalian immune system, which will eventually sequester and clear them [14], however, this can be modulated by altering the surface proteins of the phage, creating long circulating phage particles [15].

Unfortunately, there are no magic bullets, and a second primary obstacle must still be overcome before AAVP can be used clinically, namely that of intracellular host-defenses. AAVP is still a bacteria virus that has evolved to infect bacteria only with no optimized strategies to deliver genes to human cells. We previously reported that gene delivery by AAVP is enhanced by a broad spectrum of histone deacetylase (HDAC) inhibitors, such as suberoylanilide hydroxamic acid (SAHA) and trichostatin-A (TSA) [16]. HDAC inhibitors in general, and HDAC6 inhibitors specifically, have been looked at as promising tools for the development of anti-cancer drugs in their own right [17]. In our previous study [16], we included TSA as a control pan-HDAC inhibitor and reported that SAHA and TSA, both Zn^2+^ binding inhibitors of HDACs class I and II, restore gene delivery efficacy for the RGD4C-AAVP vector. Nicotinamide, a class III HDAC inhibitor, and valporic acid, an inhibitor of class I HDACs, had no effect on gene expression from the RGD4C-AAVP vector. In the present study, our aim was to investigate the novel HDAC6 inhibitor C1A, which has been shown to have anti-tumor properties [18], in combination with RGD4C-AAVP. We also included sodium butyrate (NaBu) as a widely used pan-HDAC inhibitor that we have not previously tested in combination with AAVP. NaBu is a short fatty-acid HDAC inhibitor that is structurally dissimilar to both SAHA and TSA, and acts on most HDACs but with limited activity for HDAC6, HDAC8, and class III HDACs [19]. 

Because HDAC6 is required for proliferation of melanomas [20] we used M21, a human melanoma cell line reported to express the α_v_β3 integrin receptor of the RGD4C-AAVP [11]. We compared this against the normal non-tumorigenic HEK293 cell line, as we reported that these cells express the α_v_ integrin receptors and have previously been used as a standard in vitro model for AAVP-mediated gene delivery [9]. Moreover, we included the murine B16 melanoma cells as an additional melanoma model. Malignant melanoma is an aggressive form of skin cancer with a high rate of metastasis and risk factors including intense intermittent UV exposure, family and personal history of melanoma, and phenotypic features such as fair skin type [21]. The incidence of melanoma in Europe varies by region and ranges from 6 to 19 cases per 100,000 [21]. Metastatic melanoma carries a poor prognosis with a median survival of less than 12 months even with treatment [21,22,23], and commonly metastasizes to both the skin and brain [24]. The disseminated nature of metastatic melanoma requires systemic therapy for efficient treatment.

Another motivation of testing melanoma is its accessibility in the clinic. For instance, the M13 phage (parent of RGD4C-AAVP) displaying tumor targeting ligands were administered intravenously to patients with stage IV melanoma and these accumulated in tumors upon intravenous injections, even repeatedly without unwanted side effects [25]. Subsequently, phage was recovered from every single tumor and phage recovery augmented with the increased doses. Melanoma is thus both an important therapeutic target as well as a suitable model to validate the effectiveness of C1A and AAVP in combination.

## 2. Results

### 2.1. Sensitivity of M21 and HEK293 Cells to C1A and NaBu

**Figure 1:**
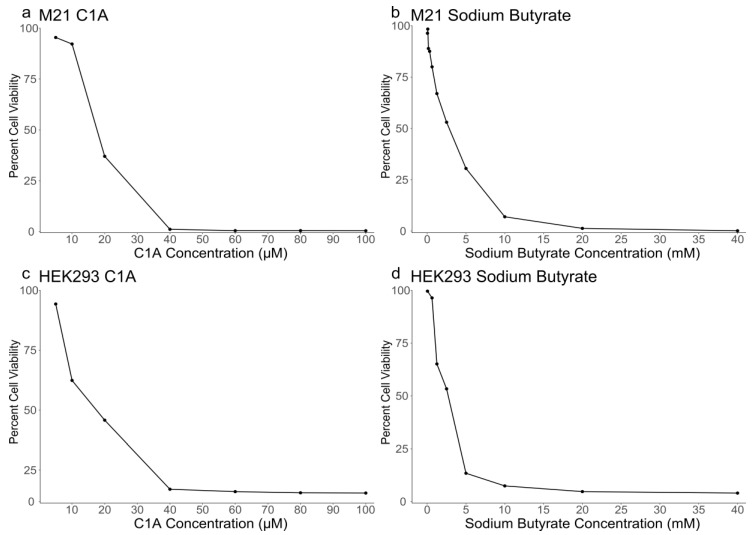
Cytotoxicity of C1A and sodium butyrate on cancerous M21 (**a**,**b**) and non-tumorigenic HEK293 (**c**,**d**) cells. Both cell lines were cultured in 96 well plates and treated with increasing concentrations of either C1A (**a**,**c**) or sodium butyrate (**b**,**d**). Cell viability percentages are relative to parallel control untreated cell cultures. The assay was repeated twice, in triplicates.

Both M21 and HEK293 cells were previously reported to express HDAC6 [20,26]. First, we determined the sensitivity of these cells to increasing concentrations of C1A and NaBu. Thus, the cytotoxicity of the drugs was investigated in vitro on M21 and HEK293 cell lines. The cells were treated with various concentrations ranging from 10 to 100 μM for C1A and 10 to 100 mM for NaBu, then compared to non-treated cells. In both cell lines, cell survival in the presence of C1A or NaBu decreased as the concentration of the drug increased (Figure 1). Median lethal concentration (IC_50_) was consistent between the two cell lines for both C1A (~18 μM) and sodium butyrate (~2.7 mM) (Table 1). However, at higher doses, HEK293 persisted in small amounts, whereas M21 were nearly entirely eradicated (Figure 1).

### 2.2. Targeted Gene Delivery to HEK293 Cells by RGD4C-AAVP in Combination with NaBu and C1A

**Figure 2:**
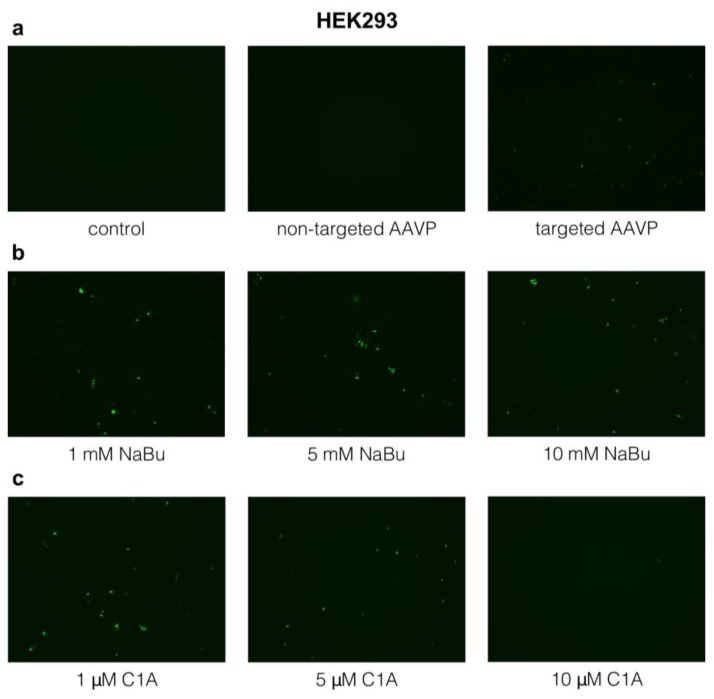
Gene delivery by RGD4C-adeno-associated virus/phage (AAVP) in vitro into HEK293 cells 6 days post vector transduction with and without adjuvants. (**a**) Control (untreated cells) and non-targeted AAVP did not show any green fluorescent protein (GFP) expression, while targeted AAVP (RGD4C-AAVP) showed some transgene expression. (**b**) Sodium butyrate (NaBu) increased transgene expression. (**c**) C1A enhanced transgene expression but did not increase with increasing doses. The highest dose of C1A examined 10 (μM) had deleterious effect on the cells. The experiment was repeated twice in triplicates and the results shown are representative of one experiment. Images are shown at 20× magnification.

These experiments were first performed to test the effects of C1A and NaBu on gene delivery by RGD4C-AAVP. Indeed, HEK293 cells have been used as an in vitro cellular model and positive control for gene delivery by targeted RGD4C-AAVP [9] as they are permissive to transduction by this vector. In this experiment, we used vector carrying the green fluorescent protein (GFP) reporter gene. Parental non-targeted AAVP vector, lacking the RGD4C ligand and expressing GFP, were applied as negative controls at the same ratio for consistency across experimental regimens even though it did not show any substantive GFP expression (Figure 2a). This was the case throughout the course of the experiment for all non-targeted AAVP variants, as we previously reported [9]). The GFP reporter was used as a simple and qualitative way to monitor AAVP-mediated cell transduction before gene delivery quantification experiments. We found that targeted AAVP, displaying the RGD4C ligand, can transduce the HEK293 cells without adjuvants, but only at relatively low levels (Figure 2a).

HDAC inhibitors had a limited effect on GFP transgene expression by targeted AAVP. The broad spectrum HDAC inhibitor NaBu only slightly increased GFP transgene expression at 1 mM concentration (Figure 2b). Therefore, we applied 5 and 10 mM concentrations despite them being over the IC_50_. However, transgene expression did not increase at higher concentrations of NaBu (Figure 2b). As with NaBu, the effect of C1A on HEK293 cells was minor but noticeable and did not seem to increase with the C1A dosage (Figure 2c).

### 2.3. Targeted Gene Delivery to M21 Cells by RGD4C-AAVP Is Improved by NaBu and C1A

**Figure 3:**
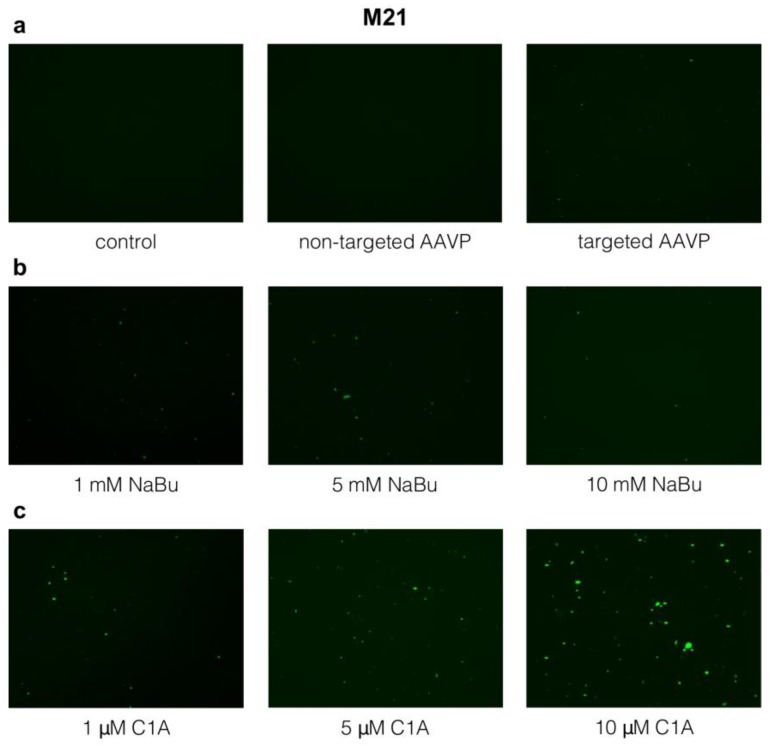
Gene delivery by RGD4C-AAVP in vitro into M21 cells 6 days post vector transduction with and without adjuvants. (**a**) Control (untreated cells) and non-targeted AAVP did not show any GFP expression, while targeted AAVP showed some transgene expression. (**b**) Sodium butyrate (NaBu) increased transgene expression in M21 cells. (**c**) C1A increased transgene expression in M21 in a dose dependent manner and more profoundly than the sodium butyrate. The experiment was repeated twice in triplicates and the results shown are representative of one experiment. Images are shown at 20× magnification.

As with HEK293, the M21 cells showed no GFP transgene expression with the non-targeted AAVP, despite being applied at the same ratio across treatment regimens (Figure 3a). Targeted AAVP displaying the RGD4C ligand could also transduce M21 cells albeit to a lesser extent than the HEK293 cells, despite being transduced with 10 times more phage transducing units (TU)/cell: 10^5^ TU/HEK293 cell vs. 10^6^ TU/M21 cell (Figure 3a).

Sodium butyrate modestly increased GFP expression in M21 (Figure 3b). As we did not see a clear change at 1 mM in our initial experiments, we also tested 5 and 10 mM concentrations despite them being over the IC_50_. Interestingly, however, C1A increased GFP expression in M21 cells, which was more pronounced than that of sodium butyrate, and the effect of increasing dosage was more readily apparent (Figure 3c). Phase contrast images of M21 cells corresponding to the experimental conditions of vector transduction at day 6, are shown in Supplementary Figure S1.

### 2.4. Quantitative Analysis of C1A and NaBu Effects on AAVP-Mediated Gene Delivery to Human Melanoma

**Figure 4:**
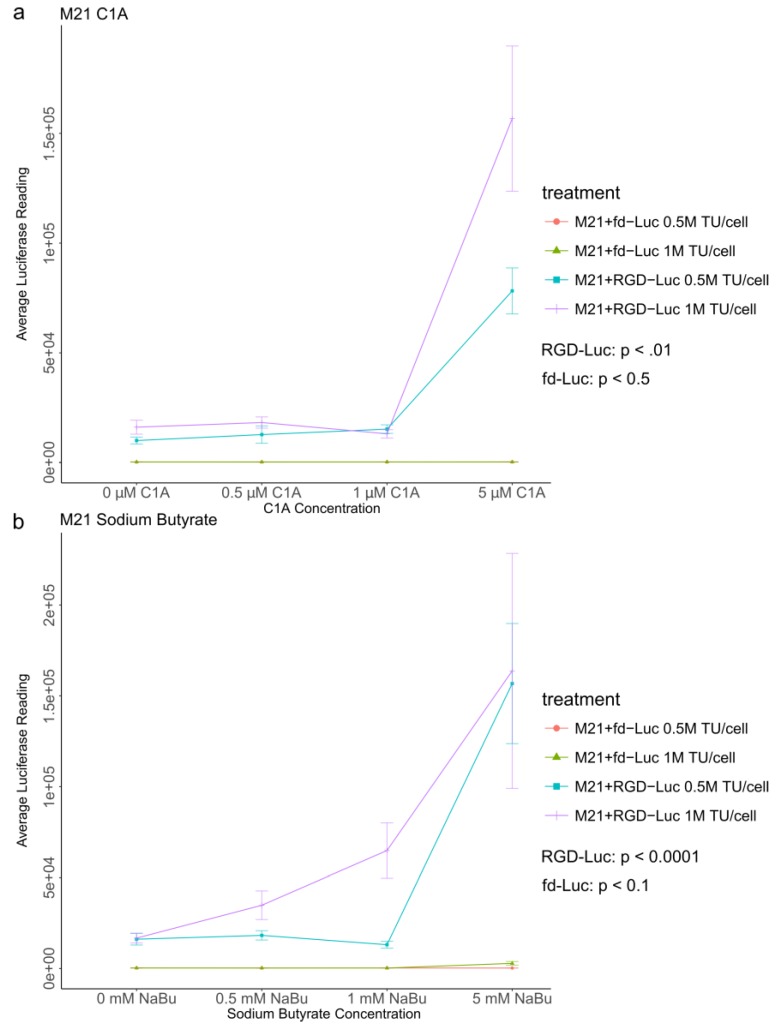
Luciferase assays with targeted AAVP (RGD-Luc) and non-targeted AAVP vectors (fd-Luc) expressing a luciferase (*Luc*) reporter gene at two different amounts of transduction with AAVP, 5 × 10^5^ and 1 × 10^6^ transducing units (0.5 million (M) TU/cell and 1 million (M) TU/cell, respectively). (**a**) M21 cells at day 4 post vector transduction, cultured with increasing concentrations of C1A. Transgene expression was dependent on targeting and increased both with dosage of C1A and AAVP TU. (**b**) M21 cells at day 4 post vector transduction and cultured with sodium butyrate (NaBu). Transgene expression was dependent on targeting and increased with dosage of sodium butyrate and with increasing AAVP TU. Experiments were carried out in triplicates and repeated twice. The vertical bars indicate standard deviation.

After showing the effect of HDAC inhibitors on AAVP targeted gene delivery to cancer using qualitative analysis of GFP expression, we sought to perform an accurate quantification of AAVP-mediated gene expression in melanoma in the presence of HDAC inhibitors. Although both 5 and 10 μM doses of the C1A increased GFP transgene expression, we tested 5 μM as a lower and safer dose that remains efficient. Thus, we used targeted AAVP vectors (RGD-Luc) and non-targeted AAVP vectors (fd-Luc) carrying a luciferase (*Luc*) reporter gene. These vectors were applied to M21 cancer cells, which were then cultured with varying concentrations of C1A (Figure 4a) or sodium butyrate (Figure 4b). In order to avoid saturation of the luciferase reporter gene which occurs relatively rapidly, we measured the luciferase expression on day 4 post vector treatment.

For RGD-Luc, C1A concentrations correlated with increased luciferase expression (*p*-value < 0.01). For the control non-targeted fd-Luc, there was no statistically significant correlation between C1A concentrations and luciferase expression (*p*-value < 0.5). Similarly, sodium butyrate concentrations correlated with increased luciferase expression of the RGD-Luc (*p*-value < 0.0001), and did not have a statistically significant correlation with fd-Luc expression (*p*-value < 0.1), the *p*-values derived from a linear regression of luciferase expression on drug concentration, that is, a drug concentration compared against no drug. These data were confirmed using two different conditions of vector transduction by applying various amounts of AAVP at 5 × 10^5^ and 1 × 10^6^ transducing units per cell for both RGD-Luc and fd-Luc with both HDAC inhibitors.

### 2.5. Quantitative Analysis of C1A and NaBu Effects on AAVP-Mediated Gene Delivery to B16 Melanoma

**Figure 5:**
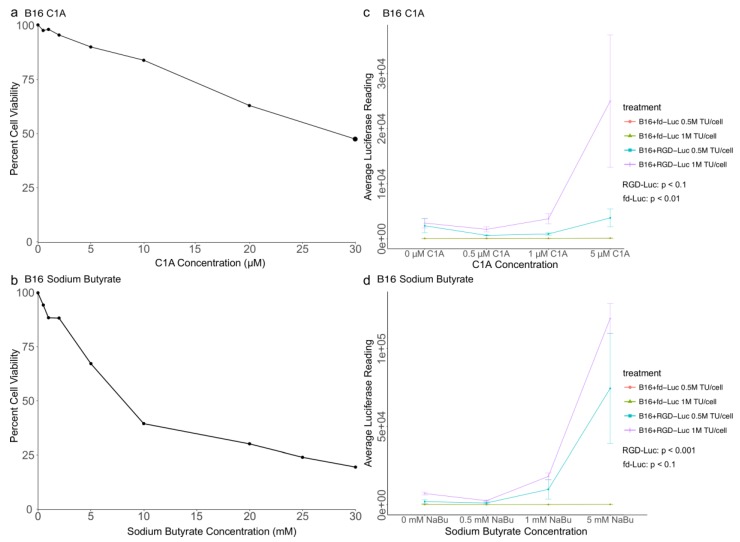
B16 cytotoxicity and luciferase assays. B16 cells were cultured in 96 well plates and treated with increasing concentrations of either C1A (**a**) or sodium butyrate (**b**). Cell viability percentages are relative to parallel control untreated cell cultures after 24 h. (**c**,**d**), luciferase assays at day 4 post transduction with targeted AAVP vector (RGD-Luc) or non-targeted AAVP (fd-Luc) expressing a Luc reporter gene at two different amounts of AAVP, 5 × 10^5^ (0.5 million (M) TU/cell) and 1 × 10^6^ (1 million (M) TU/cell) and increasing concentrations of either C1A (**c**) or sodium butyrate (**d**). Transgene expression was dependent on targeting and increased with dosage of adjuvant and with increasing AAVP TU. Experiments were carried out in triplicates and repeated twice. The vertical bars indicate standard deviation.

To confirm our findings in melanoma and rule out the possibility that the observed effects were either cell or species specific, we examined the effects of C1A and NaBu on AAVP-mediated gene delivery in B16 tumor cells, a murine melanoma cell line which is a common cellular model in melanoma research [27,28,29]. First, we examined the sensitivity of B16 cells to increasing concentrations of C1A and NaBu in order to confirm that the concentrations we tested on AAVP gene delivery to M21 cells ranged below the IC_50_ for the B16 cells (Figure 5a,b). The results were generally consistent with those of the human M21 melanoma cell line although the B16 cells were less susceptible both to C1A and sodium butyrate (Figure 5a,b). Next, we performed a luciferase experiment to carry out a quantitative analysis of the effects of C1A and NaBu on AAVP-mediated gene delivery into the B16 cells. First, no luciferase expression was detected in cells treated with the control non-targeted fd-Luc vector. Then, similar to M21, analysis of luciferase expression showed that RGD-Luc vector generated transgene expression which was significantly enhanced both by C1A and NaBu in a dose dependent manner (Figure 5c,d). Indicated *p*-values were from a linear regression of luciferase expression on drug concentrations, that is compared against no drug.

## 3. Discussion

Although in this study, simple reporter genes were used, other transgenes can be delivered by targeted AAVP, such as herpes simplex virus thymidine kinase, which makes cells susceptible to the normally non-toxic prodrug ganciclovir [30]. With its high degree of ligand-directed tissue specificity, scalability of production, and potential for systemic delivery, AAVP is already a promising vector for gene delivery to cancer cells and offers a number of avenues to further improvement.

Both the GFP microscopic imaging and luciferase quantitative assay seen here showed that the RGD4C ligand is necessary and sufficient for uptake of AAVP by cancer cells in vitro. Even in the presence of adjuvants, there is no significant increase in transgene expression with non-targeted AAVP in the examined cell lines, indicating a high degree of specificity in this treatment regimen. However, transgene expression was relatively low with the targeted AAVP, indicating a need to enhance AAVP-mediated gene delivery.

While in the absence of adjuvants, HEK293 cells appear to have higher transgene expression than M21, these M21 cells show more transgene expression in their presence. Cancer cells are generally more metabolically active than normal cells [31,32], so this expression profile was not unexpected. The HEK293 cells are more permissive but less vigorous than M21, and so HEK 293 are eventually overtaken in signal strength by the malignant cells.

HDAC inhibitors reduce the removal of acetyl groups from histones, thereby impairing chromatin condensation and transcriptional repression. The stable episomes formed by the recombinant AAV transgene cassette are also bound to histones, and therefore avoid transcriptional repression via the hyperacetylation of histones. As shown previously, and replicated here with sodium butyrate, the use of broad spectrum HDAC inhibitors boost transgene expression and improve the effectiveness of targeted AAVP. We showed here, with C1A, that selective HDAC inhibitors can replicate and even exceed this effect. HDAC6 is a predominantly cytoplasmic protein. Thus, a more plausible explanation to the increased transgene expression is the inhibition of HDAC6-mediated host-defense degradation of AAVP. HDAC6 inhibition has been reported to redirect oncolytic herpes simplex virus type 1 trafficking to the nucleus and away from the lysosomes (whereby HDAC6 mediates lysosomal degradation of viral protein), thus increasing viral replication [33]. Furthermore, bacteriophage-lambda mediated gene transfer, for instance, is enhanced by inhibiting an analogous proteasome pathway [34]. The increased gene expression of RGD4C-AAVP to melanoma M21 cells therefore perhaps represent the ability of C1A to inhibit an HDAC6-mediated host-defense against invading foreign proteins.

C1A selectively inhibits HDAC6, and so in addition to its own previously described anti-tumor properties, it appears to be comparable in effectiveness to the broad spectrum HDAC inhibitor sodium butyrate at 1000 times lower dosage. Moreover, C1A has lower cytotoxic effects than sodium butyrate relative to its boost of transgene expression. This is a particularly important consideration with the kidney derived HEK293 cells, as nephrotoxicity is a consistent problem with most chemotherapy drugs [35]. Additionally, increasing C1A dosage does not affect AAVP-mediated transgene expression in the HEK293 cells, indicating that C1A is more selective and more effective on tumor cells. Furthermore, C1A has no effect on the control non-targeted AAVP vector, lacking RGD4C, indicating that C1A does not affect the tumor targeting potential of AAVP.

Altogether, our previous and current investigation of HDAC inhibitors for their use to improve targeted AAVP-mediated gene delivery to cancer, show that a combination of AAVP with selective HDAC inhibitors has better potential than broad spectrum HDAC inhibitors, as they can be given at substantially lower doses than the pan HDAC inhibitors. This should permit achievement of both a less toxic and less costly combination of AAVP with HDAC inhibitors. Additionally, given the anti-tumor activity of C1A, the AAVP gene delivery increase by C1A, and the therapeutic ability of AAVP, this combination can have potentially synergistic interactions in tumor therapy between C1A and targeted AAVP, and offers a promising strategy that should permit both a less toxic as well as a less costly treatment of cancer. In other words, combination therapy of AAVP and C1A has potential for clinical application in cancer patients as a safe systemic treatment with fewer side effects than existing treatment regimens.

## 4. Materials and Methods

### 4.1. Cell Lines

M21 human melanoma cells were a gift from Dr. David Cheresh (University of California, La Jolla, CA, USA). HEK293 and B16F10 cells were purchased from American Type Culture Collection (ATCC) (USA). Cells were cultured in complete media consisting of Dulbecco’s Modified Eagle’s Medium (DMEM) supplemented with 10% Fetal Bovine Serum (heat inactivated to destroy complement), L-Glutamine (2 mM), penicillin (100 units/mL), and streptomycin (100 µg/mL) (all from Sigma, St. Louis, MO, USA). Cells were cultured as monolayers at 37 °C/5% CO_2_ and passaged upon reaching 80–90% confluence.

### 4.2. Phage Production

K91Kan, provided by Professors Renata Pasqualini and Wadih Arap from The University of New Mexico USA, is a kanamycin resistant strain of *Escherichia coli* and was used to produce AAVP viral stocks. These were cultured on Minimal M9 agar plates with kanamycin (100 µg/mL), which were then refrigerated at 4 °C until needed (Sigma, Gillingham, UK).

AAVP viral particles were produced in K91Kan according to previously established protocols [36]. Briefly, K91Kan was inoculated with the virus, and grown overnight at 37 °C. The bacterial supernatants were centrifuged to remove bacteria, then repeatedly purified and concentrated to collect the virus.

### 4.3. Adjuvant Treatment and Transduction

Cells were plated at approximately 40% confluence two days prior to viral transduction in multiwell plates. M21 were transduced with 10^6^ TU/cell, and HEK293 were transduced with 10^5^ TU/cell, according to previously established protocols in serum free media [36,37]. Briefly, cells were transduced for 4 h at 37 °C/5% CO_2_ and rotated every 30 min. Serum free transduction media were removed and replaced with complete media determined by the adjuvant being tested. Complete media had varying concentrations of either C1A or sodium butyrate added to it. Media were changed daily during an experiment. The volumes of vector were kept constant for each experiment and each plate. For each experiment, we used the minimum volume to cover the cells in order to increase accessibility of the phage vector to the cell surface.

### 4.4. Reporter Genes

#### 4.4.1. GFP

Cells were cultured in a 24-well plate and transduced using a 350 μL phage transduction volume as previously described with AAVP carrying the GFP reporter gene. GFP expression was examined using a Nikon Eclipse TE2000-U fluorescence microscope (Phihong Enterprise, Taiwan), and images were taken at 20× magnification.

#### 4.4.2. Luciferase

Cells were cultured in triplicate in a 96-well plate and transduced with 150 μL transduction volume as previously described with AAVP carrying the secreted lucia luciferase reporter gene (Lucia luciferase reporter gene, Invivogen, Toulouse, France). The secreted lucia luciferase was used as a simple method of quantitative analysis of gene expression while allowing easy direct detection from the cell culture medium, enabling kinetic studies from the same cells.

Lucia expression in transduced cells was evaluated using the Quanti-Luc Luciferase Assay (Invivogen, Toulouse, France). Briefly, 10 µL of cultured media were mixed with 50 µL of Quanti-Luc reagent in a 96-well white opaque microplate and incubated at RT for 2 min. Luciferase activity was quantified using a Promega GloMax^®^ (Promega, Wisconsin, USA)-Multi+ plate reader.

### 4.5. Cytotoxicity Assay

Cytotoxicity of C1A and sodium butyrate were examined. Cells were cultured in a 96-well plate and given a mock (vector free) transduction with serum free media. Cells were then cultured for 24 h with serial dilutions of either C1A or sodium butyrate. The CellTiter-Glo^®^ (Promega, Madison, WI, USA) Luminescent Cell Viability Assay was used to evaluate cell viability 24 h later. Briefly, culture media was removed, then cells were lysed with 200 µL of 1× Glo lysis buffer for 10 min at RT. 30 μL of cell lysate was transferred to a 96-well white opaque microplate and mixed with 30 μL of CellTiter-Glo^®^ substrate, then allowed to incubate for 10 min at RT. Luminescence signal was quantified using a Promega GloMax^®^-Multi+ plate reader.

### 4.6. Computer Programs and Statistical Analysis

Image capture and processing were performed using Openlab 5.0.2. (Improvision, Dundee, UK) Statistical analysis was conducted using R version 3.4.1 [38] as implemented in RStudio version 1.0.153 [39]. Graphs were produced using ggplot2 version 2.2.1 [40] as implemented in RStudio version 1.0.153.

## 5. Conclusions

Our findings show that HDAC6 is involved, at least in part, in the gene expression silencing of the RGD4C-AAVP gene delivery vector, overtime. Subsequently, we have presented a novel strategy to improve RGD4C-AAVP gene transfer to melanoma, specifically and other cancers that express HDAC6, by combining RGD4C-AAVP with selective inhibition of HDAC6 by using the C1A inhibitor. Additionally, given the selectivity of C1A and RGD4C-AAVP for cancer, this combination provides novel and suitable strategy to enhance cancer gene therapy by the RGD4C-AAVP without altering the safety attribute of the vector.

## Figures and Tables

**Table 1:** Median lethal concentration of C1A and sodium butyrate after 24 h of treatment.

Cell Line	C1A IC_50_	Sodium Butyrate IC_50_
HEK293	17.41 μM	2.7 mM
M21	18.05 μM	2.7 mM

## Supplementary Materials

The following are available online at http://www.mdpi.com/2072-6694/10/4/125/s1. Figure S1: Phase contrast images of M21 cells taken at day 6 post vector transduction with targeted AAVP or non-targeted vector. Untreated cells are shown as control.

## Abbreviations

AAV	Adeno-associated virus
AAVP	Adeno-associated virus/phage
B16	Murine melanoma cell line
GFP	Green Fluorescent Protein
HDAC	Histone deacetylase
HEK293	Human embryonic kidney 293 cells
IC_50_	Median lethal concentration, or 50% inhibitory concentration
M13	Filamentous bacteriophage
M21	Human melanoma cell line
MuLV	Murine leukemia virus
RGD4C	A double cyclic arginylglycylaspartic acid peptide (CDCRGDCFC) that targets αv integrins (principally α_v_β3 and α_v_β5)
RT	Room temperature
TU	Transducing unit
