# Supplementary material for: Selective Inhibition of Histone Deacetylation in Melanoma Increases Targeted Gene Delivery by a Bacteriophage Viral Vector
Source: Cancers (Basel). 2018 Apr 21;10(4):125. doi: 10.3390/cancers10040125 (PMC5923380; doi:10.3390/cancers10040125)
Supplement: Supplementary file 1 [file cancers-10-00125-s001.pdf]

Supplementary Materials: Selective Inhibition of Histone Deacetylation in Melanoma Increases Targeted Gene Delivery by a Bacteriophage Viral Vector

Samuel Campbell, Keittisak Suwan, Sajee Waramit, Eric Ofori Aboagye and Amin Hajitou

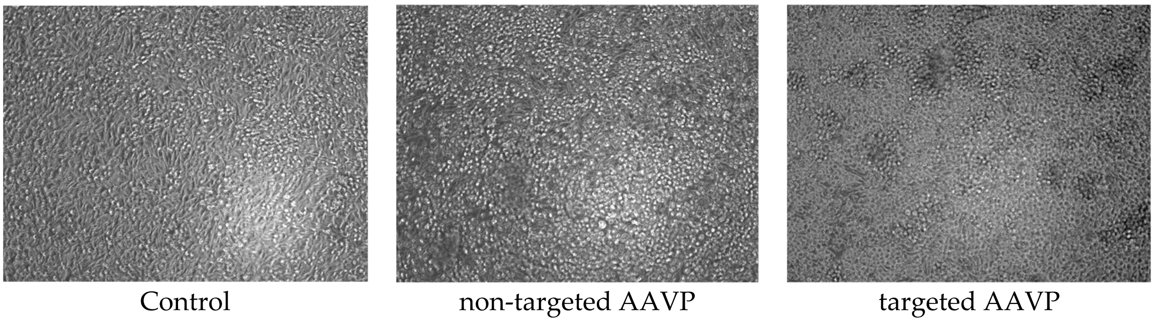

Figure S1. Phase contrast images of M21 cells taken at day 6 post vector transduction with targeted AAVP or non-targeted vector. Untreated cells were used as control. Images are shown at 100x magnification.

© 2018 by the authors. Licensee MDPI, Basel, Switzerland. This article is an open access article distributed under the terms and conditions of the Creative Commons Attribution (CC BY) license (http://creativecommons.org/licenses/by/4.0/).
